# Plant-Derived Natural Compounds in Genetic Vaccination and Therapy for HPV-Associated Cancers

**DOI:** 10.3390/cancers12113101

**Published:** 2020-10-23

**Authors:** Rosella Franconi, Silvia Massa, Francesca Paolini, Patrizia Vici, Aldo Venuti

**Affiliations:** 1 Division of Health Protection Technology, Department for Sustainability, Italian National Agency for New Technologies, Energy and Sustainable Economic Development, ENEA, 00123 Rome, Italy; 2 Division of Biotechnology and Agroindustry, Department for Sustainability, ENEA, 00123 Rome, Italy; 3 HPV-UNIT—UOSD Tumor Immunology and Immunotherapy, IRCCS Regina Elena National Cancer Institute, 00144 Rome, Italy; francesca.paolini@ifo.gov.it (F.P.); aldo.venuti@ifo.gov.it (A.V.); 4 Division of Medical Oncology B, IRCCS Regina Elena National Cancer Institute, 00144 Rome, Italy; patrizia.vici@ifo.gov.it

**Keywords:** plant molecules, HPV-related tumors, DNA vaccination, multimodal treatments, immunomodulation, immunotherapy, combined DNA vaccine/plant molecule therapy, chimeric vaccine

## Abstract

**Simple Summary:**

DNA vaccination represents a useful approach for human papillomavirus (HPV) cancer therapy. The therapeutic potential of plant-based natural compounds for control of HPV- associated cancers has been also widely explored. Genetic vaccines for HPV-associated tumors that include plant protein-encoding gene sequences, used alone or in combinations with plant metabolites, are being investigated but are still in their infancy. Main focus of this paper is to provide an overview of the current state of novel therapeutic strategies employing genetic vaccines along with plant-derived compounds and genes. We highlight the importance of multimodality treatment regimen such as combining immunotherapy with plant-derived agents.

**Abstract:**

Antigen-specific immunotherapy and, in particular, DNA vaccination provides an established approach for tackling human papillomavirus (HPV) cancers at different stages. DNA vaccines are stable and have a cost-effective production. Their intrinsic low immunogenicity has been improved by several strategies with some success, including fusion of HPV antigens with plant gene sequences. Another approach for the control of HPV cancers is the use of natural immunomodulatory agents like those derived from plants, that are able to interfere in carcinogenesis by modulating many different cellular pathways and, in some instances, to reduce chemo- and radiotherapy resistance of tumors. Indeed, plant-derived compounds represent, in many cases, an abundantly available, cost-effective source of molecules that can be either harvested directly in nature or obtained from plant cell cultures. In this review, an overview of the most relevant data reported in literature on the use of plant natural compounds and genetic vaccines that include plant-derived sequences against HPV tumors is provided. The purpose is also to highlight the still under-explored potential of multimodal treatments implying DNA vaccination along with plant-derived agents.

## 1. Introduction

Cancer is considered the leading cause of death in wealthy countries, and 15–20% of all human cancers are associated with viral infections [1,2]. Human papillomavirus (HPV) is the most common sexually transmitted virus and HPV-related cancers account for 8% of all human cancers and their annual incidence is approximately 15 per 100,000 among women and men [3,4]. The incidence of HPV-related cancers remains high despite the introduction of prophylactic vaccines [5]. Currently, commercially available prophylactic HPV vaccines are suggested for use in women up to 45 years of age, but are mainly administered in the 9–15 years’ cohort. Since most cancers develop in decades after the initial HPV infection, the impact of this vaccination program will only be seen in the long-term. Therefore, the creation of a therapeutic vaccine able to provide similar results to treatments in use in clinical practice, such as surgery or chemotherapy, represents a challenge for the eradication of HPV-induced tumors. However, no therapeutic vaccines are licensed for clinical use yet. Currently, several types of therapeutic HPV vaccines are being tested [6,7,8,9]. In this article, the status of therapeutic, “plant-inspired” HPV genetic vaccines is reviewed, together with the therapeutic potential of plant-based natural compounds. The analysis of published data demonstrated that the power of plant-based molecules/vaccines in the development of therapeutic vaccines against HPV-disease is very strong and that plant molecules may render the immune system more prone to a vaccine response.

In the past, we described that a plant extract of *Nicotiana benthamiana*, containing ectopically expressed HPV 16 E7 protein, induced a cell-mediated immune response able to protect vaccinated mice from tumor challenge, notably without any adjuvant [10,11].

This extract induced maturation of human dendritic cells (DCs) that became able to prime in vitro human blood-derived T lymphocytes from healthy individuals to induce HPV 16 E7-specific cytotoxic response [12]. A similar ability to increase immunogenicity was described for an E7 protein-based vaccine produced in the microalga *Chlamydomonas reinhardtii* [13]. A common and relevant feature of these vaccines was the intrinsic adjuvant activity.

Meanwhile, it was established that enhanced release of HPV antigens from tumor cells pretreated with chemo/radiotherapy can be modulated by plant-derived chemotherapeutic agents. Indeed, chemotherapeutic agents such as Apigenin [14] and Epigallocathenin [15] induced a powerful cell-mediated response when used in combination with DNA vaccines. On the contrary, another chemotherapeutic agent such as Saffron [16] has proven its anticancer effects used alone. Therefore, it is important to explore the mechanisms of action of both plant molecules and DNA vaccination to identify the best combination for HPV-related cancer treatment.

Beside plant molecules, plant viruses and gene sequences encoding plant proteins (including signal sequences) have been employed to improve HPV therapeutic genetic vaccine. Fusion of HPV 16 E7 gene to the Potato Virus X coat protein (PVX-CP) gene increased the rate of proteasomal degradation in transfected cells and, as a consequence, vaccine efficacy [17]. Indeed, peptides produced by the proteasomal degradation of cytosolic proteins bind to MHC I molecules and the rate of antigen degradation by the ubiquitin-proteasome pathway affects antigen presentation by MHC I, that is of pivotal importance for vaccine activity. In addition, we demonstrated that sequences derived from plant proteins improved genetic E7-based vaccine formulations [9,18]. Further, a plant signal sequence fused to synthetic E7 and L2 (i.e., the minor HPV capsid protein) genes of HPV 16 was able to elicit strong specific IgG humoral responses associated to E7 specific T-cell mediated immunity [19,20]. This chimeric vaccine, with preventive and therapeutic effects against HPV infections, offers excellent prospects for the future of DNA vaccine research. These promising efforts to create new therapeutic vaccines will help control HPV-associated malignancies alongside conventional methods of treating HPV [21].

In this review, we focus on the most relevant aspects of plant-derived compounds and genetic vaccines that might be decisive for the future development of cost-effective HPV vaccines.

## 2. HPV Carcinogenesis

The high-risk (HR) HPV types (i.e., HPV 16, 18, 31, 33, 35, 39, 45, 51, 52, 56, 58, 59, 68, 73, 82) are considered to be the main etiological agents of genital tract cancers, such as cervical, vulvar, vaginal, penile, and anal cancers, and of a subset of head and neck cancers. Among the 15 most frequent oncogenic HPV types associated with these cancers, HPV 16 is the most common and associated with the highest risk of progression to cancer [22,23,24].

The primary evidence of cancer development is HPV integration into the host genome in malignant tumors. HPV integration can take place in multiple non-recurrent regions of amplification and in flanked regions where deletions occur. There is a robust association between HPV insertional breakpoints and genomic structural alterations, which ultimately results in genomic instability, a peculiar sign of HPV-positive tumors. HPV alone is necessary but not sufficient to induce tumors, while it holds an important role in cancer maintenance. However, other genetic and epigenetic events are required for cancer development. This phenomenon may partly explain the latency period that occurs in tumor development and how persistent infection with HR HPVs is necessary for progression to high-grade lesions or cancer [25,26].

Other risk factors for progression to high-grade dysplasia and cancer include age over 30 years, infection with multiple HPV types, immunosuppression, and tobacco use [27,28].

HR HPV E6 and E7 are oncoproteins that bind and promote degradation of tumor suppressor proteins, p53 and retinoblastoma (pRb), respectively. However, HPVs interact with many other cancer-relevant pathways, even in a p53- and/or pRb-independent way. In addition, these oncoproteins may deregulate intracellular microRNA (miRNA) networks, and many HPV-altered miRNAs have been linked to carcinogenesis [29]. E6/E7 oncoproteins represent an ideal set of targets for a therapeutic vaccine against HPV-associated cancer because these proteins not only induce tumorigenesis but also are constitutively expressed in HPV-infected pre-malignant and malignant cells. Since there is evidence that regression of HPV-associated lesions is linked to the presence of a cellular, but not humoral immune response, a therapeutic vaccine able to induce a selective robust E6/E7-specific T-cell response is highly welcome [30].

## 3. HPV Vaccines

Identifying HPV as an etiological factor of cervical cancer and other HPV-associated malignancies helped in the development of immunization strategies to prevent infection and associated diseases caused by HPV. Since 2013, HPV vaccines (bivalent and quadrivalent) have been included in the national immunization programs of at least 66 nations, including North America and Western Europe, primarily [28]. Recombinant HPV virus-like particles (VLPs) are being produced at commercial level via heterologous expression of the major capsid protein L1 in yeast or insect cells [31]. From the morphology viewpoint, VLPs are similar to natural HPV virions with considerable potentialities to induce animal and human type-specific antibody responses [32].

### 3.1. HPV Preventive Vaccines

Preventive HPV vaccines aim to prevent HPV infection by inducing a neutralizing antibody response. Improved understanding of protective humoral immune response against primary HPV infection has led to the development of preventive HPV vaccines targeting L1 and/or L2 viral capsid proteins [33]. Because of the difficulties of in vitro cultivating HR HPVs and their oncogenic nature, live attenuated or inactivated vaccines could not be safely developed for humans. Therefore, studies were focused on alternative methodologies as virus-like particles (VLPs). It was demonstrated that inoculum of VLPs from L1 protein of specific papillomaviruses (PVs) could protect against PV infection [34].

Development of this technology led to the production of current VLP-based preventive vaccines targeting L1 in order to generate neutralizing antibodies against HPV. Commercially available efficacious prophylactic vaccines include the bivalent Cervarix (GlaxoSmithKline, Brentford, UK) [35] as well as multivalent Gardasil-4 and Gardasil-9 (Merck) [36,37]. The recent development of Gardasil-9 has increased preventive coverage from HPV types 6, 11, 16, and 18 to 6, 11, 16, 18, 31, 33, 45, 52, and 58 [34]. Prophylactic HPV vaccines have been shown to effectively prevent vaccinated individuals from contracting HPV infections [38] but these preventive vaccines have not been successful in treating established HPV infections [39].

More than 10 years have elapsed since HPV vaccination was implemented, and a systematic review of HPV vaccination programs in 14 countries that included over 60 million people vaccinated presented evidence of vaccine efficacy [40]. In comparison with the period before vaccine introduction, the prevalence of cervical precancerous lesions decreased by 51% among girls aged 15–19 years and by 31% among women aged 20–24 years at up to 9 years after vaccination began.

However, there is still an urgent need for the development of therapeutic HPV vaccines to tackle existing HPV infections, prevent the development of cancer, and act as immunotherapies for HPV-associated malignancies.

### 3.2. Therapeutic Vaccines

Many different technologies have been utilized to develop therapeutic vaccines and most of them target E6 and/or E7 oncoproteins of HR HPV because they are constantly expressed in HPV-associated cancer [6,7,8]. It is worth mentioning that the first HPV vaccine administered to women was a live recombinant vaccinia virus expressing the E6/E7 oncoproteins of HPV types 16 and 18 [41]. Live vector vaccines employing viruses (Ankara modified vaccine virus, TG4001 Transgene Inc. France) or bacteria (Listeria monocytogenes, ADXS11-001 Advaxis Inc, Princeton, NJ, USA) are in clinical trials with promising results (NCT03260023 and NCT02853604, respectively). Nevertheless, live vector-based vaccines pose potential safety risks, in particular in immunocompromised people [35]. Furthermore, using the same vector for repeated immunizations leads to a limitation of the immune response [35,38].

Protein- or peptide-based vaccines have been also evaluated and tested in clinical trials with some interesting outcomes for synthetic long peptide-vaccine in early stages of HPV carcinogenesis [42,43,44]. More challenging approaches such as vaccines based on dendritic cell (DC), tumor cells or adoptive T-cell therapy (ACT) have been developed but they cannot be easily performed and require specialized clinical centers [45,46,47,48]. On the contrary, technologies utilizing DNA or RNA vaccines can be easily performed and are in advanced clinical trials, as detailed in the following sections.

#### 3.2.1. DNA Vaccines

Nowadays, nucleic acid therapeutics accounts as promising alternatives to conventional vaccine approaches. Once a DNA vaccine has reached the nucleus of a myocyte, a primary keratinocyte, or a resident antigen presenting cell (APC), the expressed antigen gene is processed by cell machinery. Cross or direct priming of DC produces the presentation of the antigen within the class I or II major histocompatibility complex (MHC) on their surface [49] for immune recognition. However, this process is much more complex and further studies are needed to ensure that DNA vaccines can activate all the complex mechanism of co-stimulatory signals that lead to the activation and expansion of CD4+, CD8+, and naive B-effector cells. In particular, a therapeutic DNA vaccine must be able to generate both a CD8+ response, which directly kills infected or tumor cells, and a CD4+ helper response, which is able to increase and maintain the cytolytic response [50].

In addition, DNA vaccines are characterized by ease of production and high stability. Their safety and use in different administrations without losing their efficacy make them the ideal treatment for the control of HPV infections and associated diseases [51,52]. DNA vaccines also sustain the expression of antigens within cells for longer periods of time when compared with RNA or protein-based vaccines.

Many different HPV DNA vaccines have been constructed and proven to be active in pre-clinical models and few of them are in clinical trials (for review see [21]). In particular, VGX3100 (Inovio Pharmaceuticals Inc., Plymouth Meeting, PA, USA) is close to be used in humans. VGX-3100 is a plasmid DNA-based immunotherapy (HPV 16 E6/E7, HPV 18 E6/E7 DNA delivered intramuscularly by electroporation) under investigation for the treatment of HPV 16 and HPV 18 infection and pre-cancerous lesions of the cervix, vulva, and anus (Phase II/III) (NCT01304524 and NCT03603808) [53]. Two studies that are currently in phase III (NCT03721978 and NCT03185013) using VGX-3100 against cervical cancer show promising results.

VGX-3100 has the potential to be the first approved treatment for HPV infection of the cervix and the first non-surgical treatment for precancerous cervical lesions. VGX-3100 works by stimulating cellular and humoral responses against HPV 16 and HPV 18 E6/E7 oncogenes.

Another DNA vaccine with potential clinical use is the GX-188E (Genexine, Inc., Seongnam, Korea). This vaccine consists of a tissue plasminogen activator (tpa) signal sequence, an FMS-like tyrosine kinase 3 ligand (Flt3L), and shuffled E6 and E7 genes of HPV type 16/18. Flt3L and tpa are included in the fusion gene to promote antigen presentation and trafficking of the fused protein to the secretory pathway, respectively [54]. Recently, GX-188E was described to be highly efficacious in patients with grade 3 cervical intraepithelial neoplasia (CIN3) (NCT02139267) [55].

#### 3.2.2. RNA-Based Vaccines

The use of mRNA has several beneficial features: (i) Safety: there is no potential risk of infection or insertional mutagenesis; (ii) efficacy: some adjustments make mRNA stable and highly translatable; (iii) production: mRNA vaccines have the potential for fast, cheap, and scalable production, essentially because of the high yields of in vitro transcription reactions [56]. RNA-based vaccines are created using naked RNA replicons derived from alphaviruses to promote antigen-specific immune response. The replicon-based vectors can replicate in a wide range of cell types, with different expression of antigens. These RNA replicons are less stable than DNA. A combined approach with DNA-launched RNA replicon, termed “suicidal” DNA was developed for HPV vaccine in preclinical models [57]. The anti-tumor properties of some mRNAs expressing oncogene proteins such as E6 and E7 are now known. The therapeutic efficacy of this approach was assessed for TC-1 tumor lesions, demonstrating that the RNA-vaccine induced CD8 T cells to migrate to the tumor tissue [58]. Up-to-date mRNA-based vaccines are developed by CureVac (Tübingen, Germany) and represent a potential new approach in cancer treatment. For the first time, mRNA could be optimized to mobilize the patient’s immune system to fight cancer with a specific immune humoral and cellular response elicited by the RNActive^®^ vaccine. CureVac’s RNActive^®^ cancer vaccines (CV9103 and CV9104) have successfully completed Phase I/IIa clinical studies in prostate cancer and non-small cell lung cancer [59,60].

Recently, this technology has been used to develop prophylactic vaccines for infectious diseases such as COVID-19 due to the severe acute respiratory syndrome coronavirus 2 (SARS-CoV-2) [61]. Unfortunately, although the strategy of using RNA is so promising and inexpensive, there is still no news on clinical trials for HPV-associated diseases.

## 4. The Role of Adjuvants in Cancer Vaccines

The innate immune system has a key role in triggering an active adaptive T-cell response in the initial phase of in vivo priming [62]. Thus, many molecules that activate innate immunity and support T-cell response have been tested as vaccine adjuvants in clinical use. These adjuvants can also operate as a local depot for antigen protection from degradation [63].

Toll-like receptor (TLR) agonists, (such as CpG, imiquimod and poly I:C, that activate TLR9, and TLR7 and TLR3, respectively [64]), have been employed, as well as cytokines [65,66] and glycolipid ligands [67]. TLRs and agonists of CD40 were able to stimulate tumor specific immunity that in turn elicited cancer regression [68]. Same activity was reported for TLR3 agonists in combination with Freund’s incomplete adjuvant (IFA) or anti-PD-1 antibodies [69].

Bacteria- such as IFA and Montanide or virus-derived molecules are the most utilized adjuvants. Bacille Calmette-Guerin (BCG) is utilized as a therapeutic vaccine and its adjuvant activity is mediated mostly by TLRs. On the contrary, TLR independent adjuvant activity can be elicited by cytosolic nucleic acids secreted by bacteria. Cyclic dinucleotides (CDNs) may activate stimulator of interferon genes (STING) that activates TANK-binding kinase 1/interferon regulatory factor 3 (TBK1/IRF3), nuclear factor κB (NF-κB), and signal transducer and activator of transcription 6 (STAT6) signaling pathways causing type 1 Interferon (IFN 1) and proinflammatory cytokine activation in response to deviant host cells (danger associated molecular patterns, DAMPS) or cytosolic double stranded DNA (dsDNA) from pathogens [70,71]. Indeed, cancer vaccines with STING agonists were proven efficacious in different pre-clinical animal models and were shown to induce a marked programmed death ligand 1 (PD-L1) up-regulation, which was associated with tumor-infiltrating CD8(+) IFNγ(+) T cells. A synergistic activity with PD-1 blockade was demonstrated in poorly immunogenic tumors that were no responder to PD-1 blockade alone [72]. Association of anti PD-1/PD-L1 antibodies and STING agonists are now under study in clinical trials (NCT03172936).

Systemic adjuvants are represented by cytokines and monoclonal antibodies. However, conflicting results were reported in human cancer for cytokines [73,74,75] or antibodies [76] administration and the differences in treatment schedule may account for these results that limit their clinical use. In addition, evidence that a specific cancer vaccine adjuvant is superior to another one is lacking.

Plant extracts are emerging as new adjuvant compounds in cancer vaccines. *Nicotiana benthamiana* plant extracts as well as *Chlamydomonas reinhardtii*, a well-characterized unicellular alga, and hairy root cultures may display adjuvating activity in cancer vaccines significantly eliciting type 1 helper T cells (Th1) and cytotoxic T-lymphocyte (CTL) responses [10,11,13,77,78,79]. Moreover, plant extracts are able to exert immunomodulatory activity in vitro on DC [12]. Adjuvant activity of plant components has been also reported in studies on Zera^®^ peptide, a self-assembling domain of the maize gamma-zein seed storage protein. This peptide is able to target recombinant proteins to endoplasmic reticulum and to determine their accumulation as protein bodies (PBs). These PBs induce stronger immune responses compared to the soluble recombinant proteins. Zera^®^ peptide has been either fused or combined (i.e., mixed) to a harmless shuffled HPV 16 E7 (16E7SH) synthetic protein. Significantly higher humoral and cellular immune responses to E7 were induced either as ZERA-16E7SH fusion protein or as Zera^®^ PBs mixture with 16E7SH compared to 16E7SH alone. This effect is supposedly determined by a more efficient antigen presentation by PBs and suggests that Zera^®^ may act as an adjuvant [80].

Thus, different plant components may exert common potentiating activity on therapeutic vaccines, which further strengthens the plant-based platforms as useful tools for vaccine preparation.

## 5. Plant Metabolites Targeting HPV Tumors

Even if significant progress was made against HPV disease through preventive vaccination, and despite the success of experimental therapeutic vaccines, early and efficient treatment of HPV cancers is still a challenging issue. For this reason, an active area of research has involved and still considers plants as a source of potential pharmaceutical agents for treatment of HPV-associated tumors. A well-known example is genital warts, that can be treated with plant-based anticancer therapies, such as vincristine, vinblastine, paclitaxel, camptothecin, and podophyllotoxin [81].

Indeed, plant-derived compounds represent about 75% of the whole approved anti-tumor drugs, as either natural products themselves or as molecules mimicking or directly deriving from natural sources [82]. In many cases, plant-derived compounds can be considered an abundantly available, cost-effective source of ingredients. They can be either harvested directly in nature, or obtained from plant cell cultures.

Many plant-derived compounds possess the specificities of ideal chemopreventive agents, with no effect on normal cells, bioavailability, multiple mechanisms of action, easy manner of administration and significant cellular effects in combating oncogenesis, as they may prevent carcinogens from reaching their targets, inhibit malignant cell proliferation, to modulate tumor suppressing agents and immune surveillance [83,84].

Moreover, some of them (like polyphenols) have a prominent role in neutralizing reactive oxygen species, that are well-established messengers in intracellular signaling inducing oncogenesis and as genotoxic damage inducers [85]. As an example, high levels of 8-OhdG (8-hydroxy-2′-deoxyguanosine, one of the predominant free-radicals induced in oxidative lesions) are specific to cervical carcinogenesis and characteristic of the progression from squamous intraepithelial lesions (SIL) to invasive carcinoma [86]. In addition, lipid peroxidation and block of antioxidant functions are seen in patients with several malignant pathologies of the cervix [87,88].

Despite the significant advantages of high specificity and low toxicity of plant-derived compounds as anti-cancer agents, main drawbacks can be the rapid catabolism and the low bioavailability at the target site. Combination with existing drugs, to reach synergistic effects, or the use of nanoformulation of polyphenols, to prevent their degradation, have showed promising results [89].

Phytochemicals, as either purified and characterized entities (i.e., mainly secondary metabolites), or extracts and mixtures composed by different herbal derivatives, were, indeed, the first compounds to be used in the search of tools able to tackle cervical cancer even before its etiology was discovered and since HeLa (HPV 18 positive) cells were developed in 1952 [90,91].

It has been shown that anti-cervical cancer drugs can be found in several ethno-botanical sources and there is a wealth of plant extracts that were described to have HPV-related effects (for a very exhaustive list see [92]). Nevertheless, very often these compounds are not widely distributed in the plant kingdom and not reasonably accessible. In other cases, mixtures have no indication of effective constituents and their roles in HPV-specific cytotoxicity. Also the potential role of traditional Chinese medicine has been evaluated by in vitro and in vivo experiments with studies exploring the mechanisms of action of its active components (reviewed in [93]). All the compounds tested so far need to be screened further and on a really large scale especially in vivo and in clinical settings to finally establish their HPV-specific effects in order to establish them as useful, efficacious, cost-effective, and clinically available therapeutics. Nevertheless, the available studies give a strong idea of the variety of targets and of the potential of plant-derived compounds against HPV lesions.

In the following sections, some studies describing effects of phytochemicals or extracts on HPV cancer cells in vitro and in vivo will be described. A particular mention for those directly affecting HPV E6/E7 activity or displaying adjuvant properties in combination with chemo- or radiation therapies will be deserved.

### 5.1. HPV-Related In Vitro and In Vivo Studies Based on Purified Phytochemicals

Several in vitro studies mainly focused on purified phytochemicals and, in particular, on polyphenols, among which mainly flavonoids, as well as on other chemical species such as alkaloids, polysaccharides and protein-based entities, along with plant extracts (Table 1).

Polyphenols are a heterogeneous group of chemical substances known to have safe preventive and anticancer effects and ability to specifically target viral oncogenes [146]. It is indubitable that they are of great interest as pharmaceutical agents also against cancers with viral etiology like HPV. As an example of the interest of application of this class of molecules against cancer, the effects of different polyphenols against oral squamous cell carcinoma (both HPV+ and HPV−) has been reported in a wealth of literature (reviewed in [147]).

Anthocyanins (polyphenols, flavonoids) are mainly encountered in berries, currants, eggplant, grapes, and black rice. The feature of anthocyanins, as many tumor cells growth inhibitors, is related to their ability to induce tumor cells apoptosis and neutralize ROS [148]. In particular, anthocyanin from black rice and cyanidin 3-glucoside were found able to block the growth of HeLa cells by apoptosis mediated by Bax/Bcl-2, through a dose- and time-dependent mechanism [94].

(-)-Epigallocatechin-3-gallate (EGCG) (polyphenols, flavonoids, cathechins) extracted from green tea, can contrast tumor cell growth interfering with tumor-related angiogenesis and propensity to metastasize. EGCG was demonstrated also to have a possible gene regulatory role. In the case of HPV, it was shown in vitro that CaSki (HPV 16 positive) cervical cancer cells are guided to apoptosis by EGCG with cell cycle arrest in the G1 phase. Furthermore, in vivo anti-HPV tumor effects of EGCG were also observed [95].

The mechanism found for EGCG suppression of HPV-related cancer cell lines is the inhibition of HPV E6/E7, estrogen receptor α, and aromatase expression by apoptosis in a time-dependent manner [96,97]. Indeed, a critical role of estrogens in cervical cancer is known [149]. It was also demonstrated that different responses were found in squamous cell carcinoma and in adenocarcinoma, the latter being less responsive to EGCG inhibition [98]. In HeLa cell line, EGCG was able to repress hypoxia- and serum-induced HIF-1α and VEGF, through MAPK and PI3K/AKT [100].

In HeLa cells, EGCG abrogated HDAC1 activity, also affecting expression of retinoic acid receptor-β, cadherin 1, and death-associated protein kinase-1 [150], showing participation of these genes in cell processes strongly associated with cancer proliferation.

A genome-wide study reported that EGCG affects also DNA methylation in oral squamous cell carcinoma (CAL-27) [46]. Besides, EGCG was demonstrated to suppresses HeLa, CaSki, and C33A (HPV negative) cell growth via regulating the expression of miRNAs, suggesting as potential therapeutic targets for the control and prevention of cervical cancer [99].

Apigenin (polyphenols, flavonoids, flavones), the main plant-derived bioactive flavone, is abundant in common fruits and vegetables such as parsley and celery, onions, oranges, wheat sprouts, and chamomile [86]. Apigenin has been demonstrated to have anti-carcinogenic effects against CaSki, HeLa, and C33A cervical cancer cell lines [105]. Apigenin treatment arrested HeLa cells growth at G1 phase and consequentially induced p53-dependent apoptosis associated with increased expression of p21/WAF1, Fas/APO-1, and caspase-3 [106]. Apigenin also decreased expression of the antiapoptotic factor Bcl-2 [106]. It was also demonstrated that apigenin-treated HeLa cells can undergo modifications in cell motility and inhibition of translocation (i.e., a reduction of the invasive potential) probably by interference with gap junctions [107].

Jaceosidin (polyphenols, flavonoids, flavones) was first isolated in plants of the *Compositae* family. This compound was demonstrated to inhibit the function of E6 and E7 oncogenes by impairing binding of these oncoproteins with p53 and pRb and making them non-functional [109].

Luteolin (polyphenols, flavonoids, flavones) is most often found in leaves and its sources include celery, broccoli, green pepper, parsley, thyme, perilla, chamomile tea, carrots, olive oil, peppermint, rosemary, oranges, and oregano. It was demonstrated to induce apoptosis in HeLa cells [111]. Luteolin binds to a hydrophobic pocket at the interface between E6 and E6AP and mimics the leucines in the conserved α-helical motif of E6AP, displaying an E6 inhibitor activity [110]. Luteolin was also shown to sensitize HeLa cells to TRAIL-induced apoptosis by both extrinsic and intrinsic apoptotic pathways in an in vivo study [111].

Wogonin (polyphenols, flavonoids, flavones), accumulating in the plant *Scutellaria baicalensis*, was found to promote apoptosis through suppression of E6 and E7 and increase in p53 and pRb in SiHa (HPV 16 positive) and CasKi human cervix tumor cells [112].

Curcumin (diferuloylmethane, polyphenols, curcuminoids) is a hydrophobic polyphenol derived from the rhizome of *Curcuma* with a wide spectrum of pharmacological properties among which are anti-inflammatory and antioxidant activities by inhibiting lipo-oxygenase and cyclo-oxygenase [151]. Downregulation of HPV E6 and E7 oncogenes, NF-kB and AP-1, COX-2, iNOS, and cyclin D1 [123,124,125] represents the main feature of curcumin action affecting HeLa, SiHa, and C33A cells. In addition, growth suppression and apoptosis triggering associated with up-regulation of Bax, release of cytochrome c, and downregulation of Bcl-2 and Bcl-XL, were detected [126]. In HeLa cells, curcumin was also found to down-regulate HPV transcription, to target AP-1 transcription factor affecting expression of E6 and E7, and to block expression of *c*-fos and fra-1 [122].

The cytotoxic potential of diarylpentanoids, curcumin analogues, was evaluated in HeLa and CaSki cervical cancer cell lines as an improved alternative to curcumin. In particular, the MS17 analogue 1,5-Bis(2-hydroxyphenyl)-1,4-pentadiene-3-one exhibited cytotoxic, anti-proliferative, and apoptosis-inducing potential. Apoptosis was sustained by activation of caspase-3 activity in CaSki cells. Quantitative real-time PCR also detected significant down-regulation of HPV 18- and HPV 16-associated E6 and E7 oncogene expression following treatment [127].

Tanshinone IIA (abietane-type diterpenoid) from *Salvia* species, downregulates E6 and E7 and trigger apoptosis, inhibiting growth of HeLa, SiHa, CasKi, and C33A cells [130].

Berberine (benzylisoquinoline alkaloid) mainly accumulates in plants of the *Berberis* species and shows anti-inflammatory and anti-cancer properties with no apparent toxicity. Berberine interferes with of E6, E7, p53, pRb, and c-Fos expression, ultimately leading to the inhibition of cervical cancer cells growth [131]. Furthermore, it determines epigenetic changes and alters microtubules in cervical cancer cells [132].

Withaferin A (Steroid Lactone) from *Withania somnifera* induces apoptosis of CasKi cells through E6 and E7 repression determining inhibition of tumor growth [133].

Other secondary metabolites, together with polysaccharide, lectin, and protein/peptide fractions that are not described in the text are listed with their HPV-related activities in Table 1.

### 5.2. HPV-Related In Vitro and In Vivo Studies Based on Plant Extracts or Mixtures

Compared to purified phytochemicals, few studies are available on the anti-carcinogenic effects of crude or partially fractionated extracts or mixtures composed by different herbal derivatives.

Inhibition of AP-1 and STAT3, known to induce cervical carcinogenesis, and specific down-regulation of viral oncogenes E6 and E7 expression have been demonstrated for rhizome extracts from *Pinellia pedatisecta*, for Bryophyllin A-rich leaf fractionated extracts from *Bryophyllum pinnata*, for fruit extracts from *Phyllanthus emblica* and for oils extracted from *Brucea javanica* [139,140,141,142].

Two botanical formulations called, respectively, “Basant”, made of purified curcumin and saponins mixed to *Emblica officinalis* and *Aloe vera* extracts and *Mentha citrata* oil [143], and “Praneem”, composed by purified saponins, extracts from *Azadirachta indica, Emblica officinalis*, and *Aloe vera* mixed to *Mentha citrata* oil, have been shown able to block transfer of HPV16 pseudovirions in HeLa cells [143,152].

The anti-cancer effects of *Cudrania tricuspidata* stem extract were evaluated in HPV-positive cervical cancer cells (CaSki and SiHa cells, 2.5 × 10^5^ cells/mL) and HaCaT human normal keratinocytes. This extract showed dose-dependent cytotoxic effects in cervical cancer cells with no cytotoxic effect on HaCaT keratinocytes at concentrations of 0.125–0.5 mg/mL. The extract induced apoptosis by down-regulating the E6 and E7 viral oncogenes in SiHa cervical cancer cells. Its mechanism of induction of apoptosis was exclusively based on the increase of mRNA expression of extrinsic factors (i.e., Fas, death receptor 5, and TRAIL) and on activation of caspase-3/caspase-8 and cleavage of polyADP-ribose polymerase. No effects on intrinsic pathway molecules such as Bcl-2, Bcl-xL, Bax, and cytochrome C were observed. These results suggest that this extract can be used as a modulating agent in cervical cancer [144].

The in vitro biological activities of *Ficus carica* fruit latex were explored onto cervical cancer CaSki and HeLa cell lines. Data show that latex inhibits rapid growth and invasion and downregulated the expression of p16 and HPV onco-proteins E6, E7 [145].

### 5.3. Evaluation of Plant Compound Adjuvant Activity in Chemo- and Radio-Therapies for HPV-Associated Cancer

Although prophylactic vaccination represents the most effective method for cervical cancer prevention, chemotherapy is still the primary invasive intervention against HPV cancer lesions. It is urgent to exploit low-toxic natural anticancer drugs on account of high cytotoxicity and side-effects of conventional agents. Moreover, the resistance of cervical tumor cells to chemo- and radiotherapy is one of the crucial problems in the treatment of cervical neoplasia, leading to decreased efficacy or failure of the therapy. In this field, the combination of natural compounds with chemotherapy and radiotherapy in the treatment of cervical cancer was reported to improve in some cases sensitization of HPV cancer cells and to minimize the toxicity of these therapies (Table 2).

#### 5.3.1. Phytochemicals with Chemosensitizing Effects on Cervical Cancer Cells in Vitro

Curcumin was demonstrated to sensitize cervical cancer cells to taxol- or cisplatin-induced apoptosis by down-regulation of NF-kB [153,167]. Similarly, a class of metabolites of curcumin, tetrahydrocurcuminoids, increased the sensitivity of vinblastine, mitoxantrone, and etoposide in a drug-resistant human cervical carcinoma cell line [124].

Apigenin showed synergistic effects with paclitaxel improving apoptosis rates of HeLa and SiHa cancer cells [157]. Quercetin, saikasaponins (triterpenoid saponins from the plant *Bupleurum falcatum*), wogonin, and apigenin sensitized cervical cancer cells to cisplatin by sensitizing HPV-related cancer cells to paclitaxel-induced apoptosis through intracellular ROS accumulation [154,155,156]. Also, formononetin (an isoflavone found in a number of plants and herbs such as red clover) was found to sensitize cervical cancer cells to the anthracyclin epirubicin via ROS production [158].

The combination of tea polyphenols with EGCG and bleomycin demonstrated to have therapeutic effects on cervical cancer. Bleomycin is an anti-neoplastic chemotherapeutic used in redox-related cancer, including cervical squamous cell cancer, that cause severe side effects in normal cells such as immune system damage, pneumonitis, and pulmonary fibrosis, which are mediated by redox status disturbances. The combination therapy induced stronger cancer cell apoptosis ability than treated either tea polyphenols or bleomycin alone, by activating caspase-3, -8, -9, and up-regulating the expressions of p53 and Bcl-2 [159].

#### 5.3.2. Phytochemicals with Radiosensitizing Effects on HPV-Related Cancer Cells In Vitro and In Vivo

A few phytochemicals have shown radiosensitizing effects. Resveratrol [160], genistein [161,162,163], curcumin [164], ferulic acid (i.e., a phenolic acid abundant in plant cell walls) [165], and quercetin [166] have shown to increase the pro-apoptotic properties of ionizing radiation on cervical cancer cell lines in vitro and in vivo. A ROS-dependent mechanism has been postulated for these plant compounds. Genistein, a flavone mostly found in legumes, has synergistic radiosensitizing effects against several cancer cell types [168] being able to block the growth of CaSki cells in vitro, probably through cell cycle arrest.

### 5.4. Clinical Evaluation of Plant Compounds

Clinical trials focused on the anti-HPV carcinogenic effects of natural compounds were less frequent than in vitro and in vivo studies (Table 3). EGCG and curcumin were the most investigated compounds.

The clinical efficacy of EGCG and other green tea compounds (a combination of 200 mg EGCG, 37 mg epigallocatechin, 31 mg epicatechin) delivered orally ± vaginally to patients with HPV cervical lesions was evaluated. In this study, a 69% clearance rate was detected in the treated patients as compared with a 10% response rate in untreated controls, showing that green tea compounds can be effective in the treatment of HPV-related cervical lesions [146].

Green tea extracts components (i.e., polyphenon E, a standardized green tea extract containing 15% green tea polyphenols, and EGCG) were also tested in patients with HPV mild, moderate, and severe dysplasia. One of these studies recruited 90 patients. Components were applied as either ointment or oral administration. In particular, polyphenon E was applied as a topical ointment to 27 patients twice a week. On the other hand, 200 mg of polyphenon E or EGCG were delivered orally on a daily base for eight–twelve weeks.

Despite differences between ointment and capsules, giving indication of a higher efficacy of ointment with respect to capsule administration, overall, 69% of patients (35/51) showed a response to treatment with green tea components, compared to a 10% response rate (4/39) of untreated patients (*p* < 0.05) [169].

Evaluation of curcumin in clinical settings was started with a phase I clinical testing of oral administration of 0.5–12 mg of this compound for 3 months. The main result of this study was to define the safety of oral administration of curcumin as up to 8 mg/day and its bioavailability and efficacy in determining histological improvements in 1 out of the 4 patients [170].

Subsequently, a phase I/II clinical trial demonstrated that intra-vaginal administration of either curcumin capsules or Basant cream in HPV-infected women (without high grade CIN), was able to induce higher infection clearance rate (87.7% for Basant cream, 81.3% for curcumin capsules) than untreated patients (73.3%) [172]. Basant administration cleared HPV16 infection in 100% of patients (i.e., 11 HPV-infected women with low grade cervical abnormalities) recruited in a more recent study [143].

Intra-vaginal administration of Praneem for thirty days to 10 HPV-infected women with low-grade squamous intraepithelial lesions determined the clearance of HPV16 in 60% of patients. Another round of administration was able to induce HPV clearance in 50% of the patients that had not been able to eliminate HPV upon the first treatment [171]. It was postulated that the effect of Praneem administration could be a consequence of its microbicidal activity in the reproductive tract, able to neutralize infections considered a co-factor in HPV carcinogenesis [173].

### 5.5. Cytotoxicity of Plant Compounds: Anti-HPV Cancer Efficacy Prediction and Concerns for Healthy Cells

Previous sections mentioned a non-exhaustive list of plant compounds, extracts, and formulations of different origin and source tested against HPV cancers at various levels. Although many products are in the list, many of these entities need to be further clarified in terms of the role played in cytotoxicity in cancer once translated into clinic and of possible adverse effects in healthy cells. These tasks might be particularly complex to fulfill in the case of multi-component extracts. Indeed, the role of single constituents has to be investigated to evaluate the potential of the plant products as safe and effective anticancer agents.

It is clearly desirable that compounds showing cytotoxic activity in vitro against HPV cancer cell lines have also anti-cancer effects and strong tumor-selective action once tested in clinical trial. Unfortunately, not all the studies demonstrating in vitro efficacy of plant substances on HPV-related cell lines (e.g., HeLa, CaSki, SiHa, C33A) have been translated in in vivo or clinical studies, as already mentioned. The lack of clinical studies is clearly a limitation for the future clinical application of plant compounds. Despite this, it can be said that specifically HeLa and CaSki cells are models commonly used in in vitro cervical cancer research, since they contain the HR HPV types 18 and 16 viral genomes respectively, that are causing seven out of ten cases of invasive cervical cancers. Therefore, cytotoxicity of plant compounds, demonstrated by means of these cell lines in vitro, can be considered truly relevant in view of a clinical application as anti-HPV cancer agents. Moreover, plant products exerted cytotoxic effects on these cell models through specific targeting of E6, E7, and/or other hallmarks of HPV carcinogenesis. Thus, they have even more significance for a possible translation in clinical trial.

In view of clinical administration, special attention should be paid also to the issue of possible unwanted toxicity exerted by some phyto-compounds toward normal cells. Clearly, compounds with selective cytotoxic effects on cancer cells should be preferred. Undoubtedly, literature reporting comparative data on cytotoxicity of plant compounds on cancer and healthy cells should be expanded by further studies. Nevertheless, selective cytotoxic effects and doses on cancer cells have been already demonstrated for some purified compounds and extracts. For dietary compounds such as flavonoids, general safety and selective tumor cytotoxicity have been already well established [146]. Recently, *Ficus carica* latex was reported to inhibit the growth of CaSki and HeLa cells without a cytotoxic effect on human keratinocytes cell line (HaCaT) [145]. For other compounds, enhancement of immunity against cancer was demonstrated. In the case of a lipid-soluble extract of *Pinellia pedatisecta*, an enhancement of antitumor T-cell responses by restoring tumor-associated dendritic cell activation and maturation was shown [174]. Interestingly, in some cases, selective cytotoxic effects on HPV cancer cells have been demonstrated in association with downregulation of E6 and E7 oncogenes. If tumor-specific targeting is demonstrated for a certain compound in vitro, a strong clue for selectivity toward tumor cells in clinical translation is given implicitly. Plant products such as curcumin have shown selective cytotoxicity for HPV 16- and HPV 18-infected cells and induction of apoptosis in cervical cancer cells by downregulation of E6 and E7 oncogenes and of tissue-specific viral gene expression ([96,97,109,110,112,125] Table 1). As another example, the anti-cancer potential of *Cudrania tricuspidata* stem extract was investigated both in HPV-positive cervical cancer cells and in normal HaCaT keratinocytes. The extract showed significant dose-dependent cytotoxic effects in cervical cancer cells and no cytotoxicity on HaCaT. In addition, it was demonstrated that the extract-related apoptosis was induced by down-regulating the E6 and E7 viral oncogenes [144].

A strategy to avoid possible adverse effects in normal cells and to improve bioavailability of plant compounds might be to induce a confined action by tumor-targeting strategies. Nanoparticles of different types, such as multi-functionalized, magnetic or solid lipid particles, dendrimers, liposomes and micelles, some of which are already FDA approved, have been used for targeted delivery of plant compounds without disturbing the physiology of normal cells [175]. Naringenin, generally present in citrus and grapes, inhibits proliferation through cell cycle arrest at the G2/M phase and induction of apoptosis in human cervical SiHa cells. Mainly because of poor bioavailability and instability of the molecule, studies were carried out on naringenin-loaded nanoparticles that demonstrated advantages over free naringenin in HeLa cells through dose-dependent cytotoxicity, apoptosis, reduction of intracellular glutathione levels, alterations in mitochondrial membrane potential, increased intracellular reactive oxygen species (ROS), and lipid peroxidation levels [103] (Table 1). Nanoparticle-targeted delivery would offer also the advantage of multiple product loading (i.e., plant-based drugs along with synthetic drugs) beside possibly contributing to improve efficacy and decreasing unwanted toxicity. In conclusion, efficient targeting strategies accompanied by good toxicology studies could represent the future arena of research in the field of clinical application of plant products for anti-HPV cancer therapies.

## 6. Improving DNA Vaccine Effectiveness by Plant-Derived Solutions

Adjuvant activity is a conceptual issue that, in a broad sense, can be applied to any procedure/compound able to improve vaccine effectiveness. Plants and derived molecule/sequences can offer some solutions based on what we already know about their anti-cancer and apoptotic activity as well as pro-immune properties. A summary of these activities is reported, highlighting the possible adjuvant effect for HPV DNA vaccines.

### 6.1. Improved HPV Genetic Vaccines Including Plant Immune-Modulating Sequences

Although not commercially available yet, genetic vaccination represents a convenient platform with respect to traditional approaches that involve the production and purification of proteins or components of the pathogen. Owing to the ease of preparation, intrinsic safety and stability, DNA vaccination is useful for the quick assay of new synthetic immunogens as well as ensuring the induction of antibodies against conformational epitopes of interest.

Many strategies have been developed to enhance DNA vaccines effectiveness including codon optimization, particular methods of transfection (i.e., electroporation), addition of adjuvants or genetic fusions with immune-stimulating sequences, combination with heterologous boosts, etc.

Several genetic vaccines are in an advanced clinical trial phase for the treatment of HPV-associated malignancies and represent a potential additional weapon to those already available, like chemotherapy and radiotherapy. In this case, improvements of HPV genetic vaccines have focused on (1) increasing DC uptake of HPV antigens, (2) DC processing and presentation of HPV antigens, and (3) enhancing DC and T-cell interactions [21].

The fusion of HPV antigens with immunostimulatory sequences to achieve improvement in their “visibility” to the immune system, has been an approach very often explored in the development of experimental therapeutic vaccines [20]. Nevertheless, the search for innovative immunostimulatory sequences, with increased safety for clinical use, is still an open field. In fact, it is mandatory to avoid possible autoimmune responses induced by proteins of human origin (such as Hsp70 or calreticulin) or weakened immune responses (as in the case of using tetanus toxoids in already vaccinated people).

In an attempt to potentiate a genetic vaccine for HPV, the attenuated E7 gene (E7GGG) of HPV 16 was fused with the gene encoding the coat protein of a plant virus (Potato Virus X, PVX) [16]. This protein had already been used as a carrier able to increase CD4+ T-cell immune response (by “linked T-cell help”) and to enhance immunogenicity of silent or poor determinants [176,177].

It was subsequently shown that the inclusion of sequences deriving from plant proteins and peptides with immune-modulatory and anti-cancer activity [178] can potentiate the activity of HPV genetic vaccines. The rationale is that, since in mammals and plants the general mechanisms underlying the innate immune response are highly conserved [179], some plant defense proteins may have an effect also on tumors by modulating innate immune functions and, consequently, also the adaptive response. Some of these plant proteins could also stimulate specific cell-mediated immunity toward tumor antigens, a crucial step for cancer resolution. These plant proteins would therefore behave as adjuvants enhancing the specific immune response toward an antigen.

Among these proteins, some “ribosome inactivating protein” (RIPs) might be included. RIPs show a regulatory and defensive role against pathogens and accumulate in various organs of many plant species [178,180,181]. They are potent inhibitors of protein synthesis (through N-glycosidase activity on rRNAs). This was the first biological feature extensively studied and clinically exploited for the development of selective cytotoxic agents (“immunotoxins”) against tumor, immune, or nerve cells. Nevertheless, other biological properties of RIPs, independent of catalytic activity, could prove useful for the design of anticancer vaccines. Among these activities we find the ability to modulate the non-specific and innate immune functions of NK cells [182], CD4+ and CD8+ T cells, and/or cytokine production [183,184] inflammation [185], and apoptosis through multiple pathways [180,186] that have been shown to lead to anti-tumor properties in vivo [184].

Our results indeed support this hypothesis. We demonstrated for the first time that a DNA vaccine involving the fusion of the HPV 16 E7GGG gene with saporin (SAP), a RIP found in *Saponaria officinalis* but rendered no longer catalytically active through mutagenesis (SAP-KQ), potentiates antitumor activity against E7-expressing tumors. The anti-tumor activity was associated with enhanced antibody and cell-mediated immune responses and antigen-specific delayed-type hypersensibility (DTH) [18].

SAP-KQ/E7GGG fusion proteins may undergo rapid degradation via the ubiquitin-proteasome pathway, as postulated also in the case of coat protein of PVX fused to the C-terminus of E7GGG [17]. Indeed, the A-chain of type II RIPs, once entered the cytosol, is subjected to an efficient protein quality control. Upon interaction with lipid cell membranes, they are possibly recognized as misfolded proteins, ubiquitinated and then targeted to proteasomes for elimination. This is particularly true for RIPs with a high lysine content [187] like saporins, where 10% of the residues is represented by this amino acid. Ultimately, this process could improve the processing of the E7GGG antigen fused to the SAP-KQ which in turn would result in an improvement in the activity of the chimeric vaccine compared to E7GGG alone.

Then, we designed a novel genetic fusion vaccine comprising synthetic genes derived from the E7 and L2 proteins of HPV 16. Here, a signal peptide derived from a plant protein was fused upstream the N-terminal portion of the papillomavirus fusion antigen [19]. Signal sequences (ss, or signal peptides) are short peptides (about 20–30 residues) that influence the targeting pathway of a protein and promote protein secretion or specific post-translational modifications. As a result, ss from highly secreted proteins have been used to enhance protein expression levels of recombinant proteins in different cell systems. Other groups described the possibility to enhance DNA vaccines efficacy by using signal sequences that generally derive from mouse [188,189] or human pathogens [190] and there is no doubt that plant-derived sequences pose less safety concerns.

In a previous work, we had shown that the ss of the polygalacturonase-inhibiting protein (PGIPss) from *Phaseolus vulgaris,* was able to target the HPV 16 E7 protein to the plant secretory compartment, enhancing the E7 protein expression level at least five-fold compared with the unfused version of the antigen [11]. Starting from the observation that ss properties are conserved from bacteria through eukaryotes, we explored the ability of the plant-derived PGIPss in increasing the efficacy of HPV DNA vaccines. We produced a prototype DNA vaccine where the PGIPss was fused upstream of the harmless HPV 16 E7 antigen (named E7GGG or E7*). The PGIPss-E7GGG fusion was able to modify antigen compartmentalization and/or processing in transfected HEK-293cells, promoting E7 protein secretion in the culture medium, and demonstrating its ability to affect the fate of a heterologous protein in mammalian cells [19]. Furthermore, even though secretion was not observed in the culture medium, PGIPss modifies the processing of other constructs like PGIPss-E7GGG-CP, where the E7GGG gene is fused to the PVX coat protein.

**Figure 1:**
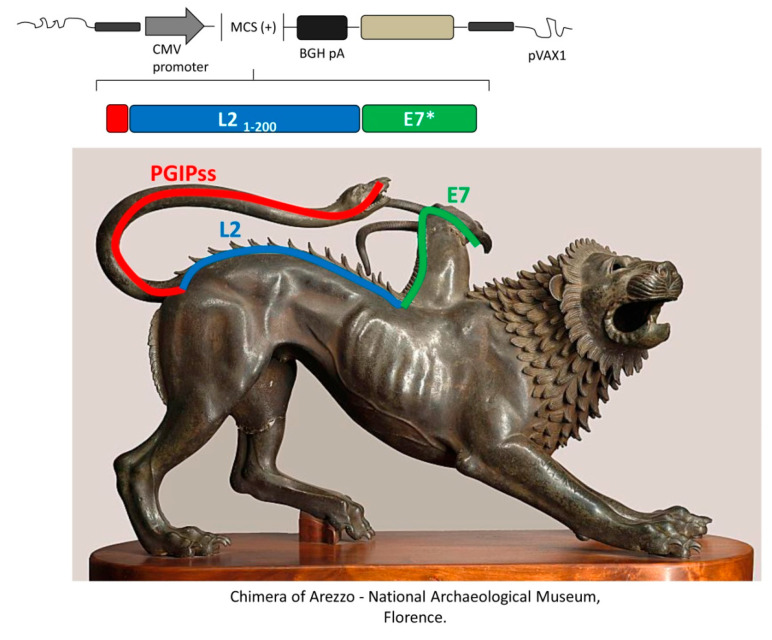
Representation of the recombinant gene deriving from the fusion of the sequences encoding HPV antigens (attenuated E7 oncoprotein and part of the L2 structural protein) and a plant signal sequence introduced into the mammalian vector pVAX1. Because of the different origin of the genes and the immunological “dynamics” conferred to the antigens by the fusion, the representation includes an iconographic comparison with the legendary “Chimera” from Greek mythology. Chimera was a hybrid creature, offspring of the giant Thyphon and of the half-woman, half-snake Echidna, incorporating a lion’s head with a goat rising from its back and a snaky tail: the different natures indicate the approach of using one nature to obtain a result in the other. CMV: cytomegalovirus promoter; BGH pA: bovine growth hormone poly-adenylation signal; PGIP*ss* (red box/line): signal sequence of the polygalacturonase-inhibiting protein of *Phaseolus vulgaris* gene; E7* (green box/line): attenuated E7 gene of HPV 16 (also named E7GGG); L2_1–200_ (blue box/line): nucleotide sequence corresponding to amino acids 1–200 of the L2 minor capsid protein of HPV type 16.

With the idea to develop a HPV preventive/therapeutic vaccine, the DNA sequence for PGIPss was fused upstream to a synthetic codon-optimized gene consisting of a cross-reactive epitope of the L2 protein (first 200 aa; L2_1–200_) and E7GGG of HPV 16, and cloned in a mammalian expression vector (pVAX1) (Figure 1). The chimeric DNA vaccine (pVAX-PGIPss-L2_1–200_-E7GGG) was delivered in C57BL/6 mice according to a prime/boost schedule, implying the use of electroporation (EP) after intra-muscular immunization.

The immunization protocol, performed together with EP, induced a long-lasting humoral IgG immune response against L2_1–200_ and E7 that persisted for at least six months upon immunization in the mouse model used together with a cell-mediated immune response [19]. Electroporation, indeed, represents an approach, consolidated by recent clinical studies, to increase the effectiveness of DNA vaccines owing to its ability to increase cell permeability with a consequent increase in protein expression level and a better immune response [53,191].

Furthermore, the new DNA vaccine was able to determine an effective tumor regression in vivo in two mouse models: the TC-1 subcutaneous model in C57BL/6 mice and the AT-84 E7 orthotopic oral model in C3H/HeJ mice [192]. The AT-84 E7 cell line was derived from AT-84 cells, that generate a spontaneous oral squamous cell carcinoma in C3H mice [192]. The natural history of the tumor AT-84 and its response to therapy resemble human oral cancer, thus allowing the study of local invasion in a more clinically relevant site. The C3H mice accept AT-84 HPV16 E7 cell grafts without immunosuppression; the derived tumors maintain stably the oncogene expression (as it happens in HPV-related human oral cancer) and grow quickly, allowing fast testing and prediction of therapy effectiveness and of treatment schedules feasibility within few weeks. In addition, we have also developed a rapid and easy way to study in vivo tumor growth by using luciferase reporter gene (bioluminescent AT-84 HPV-16 E7-Luc model) and optical imaging [193]. In vivo imaging studies provide information that cannot be obtained from *post-mortem* analysis alone, representing complementary approaches for monitoring tumor progression and treatment response in orthotopic preclinical studies. In addition, the bioluminescent AT-84 HPV-16 E7-Luc model, generates data that can be compared with those obtained by caliper measurements and allows earlier cancer detection (it has been shown that tumor mass is measurable by luminescence at day 12 when a palpable tumor is still hardly detectable) [192].

The pVAX-PGIPss-L2_1–200_-E7 DNA vaccine was delivered intra-muscle with EP in two different animal models of HP-associated tumor, resulting in a dramatic reduction of tumor growth [19]. Antitumor activity was further investigated showing that PGIPss-L2_1–200_-E7 administration induced a specific cell-mediated immune response against HPV E7 tumor antigen. Further, the ability of the PGIPss to evoke antibody response in mammalian cells was confirmed in this system [20].

As far as we know, this represents the first demonstration that a plant-derived signal sequence has biological activity in mammalian cells, increasing the immunogenicity of an antigen of interest. The chimeric PGIPss-L2_1–200_-E7 genetic vaccine represents a promising candidate against HPV-associated cancers and opens novel perspectives in the design of vaccines for other antigens and/or for different pathogens, in particular against infections where a fast and protective immune response is required (as in the current SARS-CoV-2 pandemic). Moreover, these vaccines, which can be easily produced on an industrial scale, under good manufacturing practices (GMP) conditions, represent safer vaccines as they do not involve the production of chemo/cytokines (which could induce secondary responses) or animal antigens (which could cause autoimmune cross-reactive responses).

### 6.2. Combinations of Plant Molecules with HPV DNA Vaccination

The combination of chemotherapy with immunotherapy (including DNA vaccination) might represent a potential strategy for cancer treatment because certain chemotherapy-based cancer treatments may activate the immune system against the tumor through several molecular and cellular mechanisms and reduce tumor [194].

It has been described that some natural molecules have the ability to increase vaccine-induced immunity against cancer. Furthermore, bulky tumor growth was inhibited by EGCG in combination with a genetic vaccine [15] while a multimodal treatment against progressive tumors based on apigenin and genetic vaccines demonstrated immunotherapeutic effects [14].

Nevertheless, very few works describe the effects of combined used of natural compounds with HPV genetic vaccination.

The immunomodulatory properties of *G. uralensis* polysaccharides were investigated in combination with HPV DNA vaccination [195]. *G. uralensis* polysaccharides injection had no side effect on mice, enhancing the immunity of mice and the antigen-specific cellular and humoral immune responses induced by HPV DNA vaccine.

Saffron (*Crocus sativus L.*) and its components, like monoterpene aldehyde and carotenoids derived from dry stigmas, have been suggested as favorable candidates for cancer prevention. The potential use of saffron derivatives such as extracts or purified components-carotenoids derived from dry stigmas of pure saffron alone or combined with genetic vaccination, was investigated on HPV-related experimental tumor [16]. The in vitro cytotoxic and apoptotic effects of aqueous saffron extract and its components picrocrocin, a monoterpene aldehyde and crocin, a natural carotenoid, were assessed in malignant (TC-1) and non-malignant (COS-7) cell lines. Unlike most carotenoids, that have a limited therapeutic use because of their insolubility in water, glycosylated molecules like crocin and picrocrocin (due to their glycosylated state) are soluble and highly cytotoxic on malignant cells; they therefore represent the most appropriate saffron derivatives for cancer treatment.

The anti-tumor activity of a genetic vaccine candidate and saffron components were evaluated in vivo; mice were challenged subcutaneously with TC-1 tumor cells and on day 3 and 17 they were immunized with E7-NT (gp96) DNA. Crocin, picrocrocin, and saffron extract were given orally at the time of the initial DNA immunization and for the next 16 days. While the multimodal treatment using DNA vaccine along with picrocrocin augmented the anti-tumor effects of picrocrocin, the combination of DNA vaccine with saffron extract and crocin at certain concentrations could not potentiate protective and therapeutic effects compared to mono-therapies for the control of TC-1 tumors. In particular, oral administration of crocin resulted in complete tumor regression, while it did not increase DNA vaccine-mediated anti-tumor effects. These data are apparently in contrast to those from other studies about combination of chemotherapy with EGCG [15], apigenin [14], and cisplatin [196] followed by immunotherapy with DNA vaccination. This highlights that, for proper evaluation of synergist effects of chemo-immunotherapy, the selection of the optimal dose and treatment schedule still represents a critical challenge to overcome [194,197].

## 7. Future Perspectives and Clinical Translation: What Is Needed

Together with evidences of efficacy and safety, the pharmacological use of plant-derived compounds could benefit, in many cases, from their wide availability in nature or by in vitro plant cell/tissue cultures. Certainly, chemically defined and purified entities are more suited and have higher potential as therapeutic compounds against cancer (and HPV-associated pathologies are no exception). All other plant derivatives, such as extracts and mixtures, may suffer from batch to batch variation.

In addition to this, studies to further investigate the encouraging promises of plant-derived compounds and formulations in advanced clinical trial phases, having large numbers of subjects, are needed. These studies should open the way to a deeper evaluation of the synergistic effects of plant compounds with canonical HPV tumor therapy approaches in combinatorial schedules, probably the most feasible and intriguing application of plant-derived compounds in this field.

The use of therapeutic vaccines against HPVs has been shown to induce regression of precancerous lesions of cervix and produce some clinical benefits in cancer patients. The immunosuppressive effects of the cancerous microenvironment can be attenuated by multimodal therapeutic approaches by combining the therapeutic vaccine with radiotherapy, chemotherapy, immunomodulators, and immune checkpoint inhibitors. In fact, experimental clinical evidence has clearly highlighted a synergistic action of these combinatorial approaches [8].

Therapeutic vaccines for pre- and neoplastic lesions of the uterine cervix are becoming a reality and the improvement of all therapeutic strategies associated with a multimodal approach opens a new scenario in the treatment not only of cervical cancer and pre-neoplastic lesions but also of other HPV-associated tumors. Despite to date, no therapeutic vaccine has been approved for clinical use in the treatment of HPV infections and related malignancies, it should be noted that at least some DNA vaccine candidates such as VGX-3100 and GX-188E or ADXS11-001 bacterial vector vaccine are in phase III clinical trials demonstrating that a therapeutic vaccine is in the near future (see NCT03185013, NCT02139267, and NCT02853604, respectively). In addition, therapeutic vaccines could be produced with plant expression systems that make production less expensive, expanding the possibility of their use also in low-middle-income countries where the disease burden is greater. However, these types of vaccines are still in an early stage of experimentation which makes them currently unavailable for clinical translation.

Even for DNA vaccines, their clinical use still requires a better knowledge of the mechanisms through which they are able to induce specific immune responses in vivo.

DNA vaccines can be introduced into the body by intramuscular, intradermal, or mucosal delivery through a variety of technologies, and most of them have been shown to be safe for humans despite causing varying degrees of discomfort. Furthermore, such therapeutic vaccines should not cause the activation of the immunosuppressive population of regulatory T lymphocytes (Treg). To achieve these results, vaccines and in particular DNA vaccines need to be associated to adjuvating procedures, including modification of antigen by fusion with other molecules or modifying the fate of antigen processing or host immune response (i.e., activating DC).

In an effort to make easier the transfer of adjuvated DNA vaccine from pre-clinical studies to clinical trials, it should be conceivable to use compounds and technologies already used in humans for other clinical indications. In this sense, building a DNA genetic vaccine using vectors already used in humans and with few antigen modifications can have a preferential route for their use. However, it is also important to address concerns regarding the potential for oncogenicity associated with administration of oncogenes (E5, E6, or E7) as DNA vaccines into the body. In response to that, all oncogenes in DNA vaccine are harmless version or shuffled epitopes [17,198].

If we can assume that DNA vector and modified antigen sequences are relatively safe, the same cannot be said for adjuvant activities. Such activities often involve molecules interfering with multiple pathways. This makes it difficult to directly use adjuvants in human experimentation without going through long toxicology phases in animal models. In this panorama, plant-based compounds, often derived from common food products (i.e., saffron) offer an undoubted advantage regarding their non-toxicity and, in addition, they often have a millennial use in traditional medicine. Other natural products like apigenin were already tested in several clinical trials and proven to be safe in humans [199,200].

Therefore, the study of plant compounds with adjuvant activity (especially, but not only) for genetic vaccines is a field of research that requires expansion in order to obtain vaccine products that can be used more quickly in humans. Finally, these studies will also be the basis for helping Regulatory Agencies dealing with drugs for human use by providing the necessary knowledge for an assessment of their human use.

## 8. Conclusions

Many efforts have been made to find and produce DNA therapeutic vaccines against HPV-associated lesions and cancers. The most effective vaccines in pre-clinical animal models are in clinical trials and two of them (VGX-3100 DNA and ADXS11-001) are in phase III clinical trials with promising results. Nevertheless, these vaccines are more effective in pre-cancerous lesions (i.e., CIN 2/3) than in cancer indicating that there is need of adjuvants to overcome the immunosuppressive tumor microenvironment. Plant sequences and also other genes from the “green world,” such as plant-virus genes and signal sequences could be the answer considering their safety and avoidance of any autoimmune or pathogenic response.

**Figure 2:**
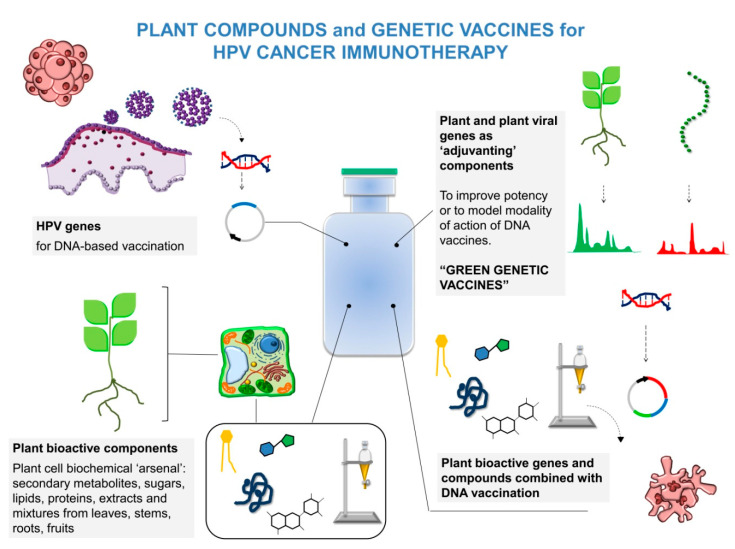
Possible new strategies against HPV tumors implying DNA vaccines, plant compounds, and plant gene sequences.

In addition, plant compounds that directly abrogate HPV E6/E7 activity are feasible candidates for a HPV phytotherapy, as they affect the major HPV “oncoplayers.” Purified phytochemicals, due to easy extraction and batch to batch consistency, will offer major perspectives to a comparatively safer alternative/combinational approach to HPV current therapy, once their efficacy in clinical trials will be confirmed. A summary of these new therapeutic strategies implying interconnections among DNA vaccines, plant compounds, and plant genes is depicted in Figure 2.

Thus, more studies are to be performed to check the effectiveness of these new therapeutic strategies including combination with existing approaches. Nevertheless, preliminary results open new horizons for the therapy of HPV-associated cancers. In addition, other tumors, where patient-specific neoantigens are detectable as a consequence of tumor-specific mutations, might benefit from these approaches [201].

## 9. Patents

Franconi R, Massa S, Venuti A. (2016). Plant protein signal sequence as adjuvant in DNA vaccines. Italian patent 102016000131935, PCT/IT2017/050008, WO 2018/122885 A1. European Patent Application 17826321.6, publication number EP 3,562,504 (EP’504).

## Figures and Tables

**Table 1:** Plant phytochemicals and extracts in relation with their effects on human papillomavirus (HPV) cancer in vitro and in vivo.

Compound Type	Phytochemicals	Study Type	Cell Type	Activity	Mechanism of Action	References
Purified	Black rice anthocyanin and cyanidin 3-glucoside	In vitro	HeLa	Inhibition of proliferation and induction of apoptosis	Dose- and time-dependent by apoptosis induction through Bax/Bcl-2	[94]
Polyphenols (Flavonoids, Anthocyanins)						
Purified Polyphenols (Flavonoids, Cathechins)	Epigallocatechin-3-gallate (EGCG)	In vitro and in vivo	CaSki	Inhibition of proliferation and induction of apoptosis	Dose-dependent apoptosis induction through arrest of cell cycle in the G1 phase.	[95]
					Possible gene regulatory role	
		In vitro	HeLa	Inhibition of proliferation and induction of apoptosis	Inhibition of HPV E6/E7 expression and of estrogen receptor α and aromatase by a time-dependent manner mediated by apoptosis	[96,97]
		In vitro	TCL-1 (HPV-immortalized cervical epithelial cells)	Inhibition of proliferation (adenocarcinoma less responsive to EGCG growth inhibition) and apoptosis induction	Dose-dependent increased expression of p53 and p21	[98]
			Me18			
			HeLa			
		In vitro	CaSki	Suppression of growth	Time- and dose- dependent, possibly via regulating the expression of miRNAs	[99]
			HeLa			
			C33A			
		In vitro	HeLa	Repression of hypoxia- and serum-induced HIF-1α and VEGF	Via MAPK and PI3K/AKT pathways	[100]
Purified Polyphenols (Flavonoids, Flavanones)	Naringenin	In vitro	SiHa	Inhibition of proliferation and induction of apoptosis	Cell cycle arrest at the G2/M phase and induction of apoptosis	[101]
		In vitro	HeLa	Inhibition of proliferation and induction of apoptosis	Reduced expression of NF-κB p65 subunit, COX-2, and caspase-1	[102]
	Naringenin-loaded nanoparticles	In vitro	HeLa	Inhibition of proliferation and induction of apoptosis and cytotoxicity	Dose-dependent cytotoxicity, apoptosis, reduction of intracellular glutathione levels, alterations in mitochondrial membrane potential, increased intracellular ROS and lipid peroxidation.	[103]
	Hesperetin	In vitro	SiHa	Reduction in cell viability and induction of apoptosis	Increased expression of caspases, p53, Bax, and Fas death receptor and its adaptor protein	[104]
Purified Polyphenols (Flavonoids, Flavones)	Apigenin	In vitro	CaSki HeLa C33A	Inhibition of proliferation and induction of apoptosis	G1 phase growth arrest through p53-dependent apoptosis and increased expression of p21/WAF1, Fas/APO-1 and caspase-3.	[105]
					Decreased expression of Bcl-2	
		In vitro	HeLa	Modifications in cell motility, inhibition of translocation	Interference with gap junctions	[106,107]
	Daidzein	In vitro	HeLa	Inhibition of proliferation	Cell cycle, cell growth, and telomerase activity alterations	[108]
	Jaceosidin	In vitro	SiHa Caski	Inhibition of the function of E6 and E7 oncogenes	Impairment of binding to p53 and pRb	[109]
	Luteolin	In vitro	Caski E6/E7 immortalized human foreskin keratinocytes (HFK) primary HFKs	E6 inhibition	Binding at the interface between E6 and E6AP mimicking leucines in the conserved α-helical motif of E6AP	[110]
		In vivo	HeLa	Induction of apoptosis	TRAIL-induced apoptosis by both extrinsic and intrinsic apoptotic pathways	[111]
	Wogonin	In vitro	SiHa CasKi	Induction of apoptosis	Suppression of E6 and E7 and increase in p53 and pRb	[112]
Purified Polyphenols (Flavonoids, Flavonols)	Quercetin	In vitro	HeLa	Induction of apoptosis Induction of mitochondrial apoptosis	G2/M phase cell cycle arrest, apoptosis, inhibition of anti-apoptotic AKT and Bcl-2 expression	[113]
	Kaempferol	In vitro	HeLa	Inhibition of proliferation	G2/M phase growth arrest, decrease of cyclin B1 and CDK1, inhibition of NF-kB nuclear translocation, upregulation of Bax and downregulation of Bcl-2	[114]
	Fisetin	In vivo/in vitro	HeLa	Inhibition of proliferation and reduction of tumor growth by apoptosis	Apoptosis due to activation of the phosphorylation ERK1/2, inhibition of ERK1/2 by PD98059, activation of caspase-8/caspase-3 pathway	[115]
Purified Polyphenols (Flavonoids, Phenolic Acids)	Gallic acid	In vitro	HeLa	Induction of apoptosis and/or necrosis	ROS increase and GSH depletion	[116]
Purified Polyphenols (Flavonoids, Stilbenes)	Resveratrol	In vitro	SiHa HeLa	Inhibition of proliferation, induction of autophagy and apoptosis	Cathepsin L-mediated mechanism	[117]
		In vitro	HeLa	Suppression of invasion and migration	Generation of ROS	[118]
		In vitro	SiHa HeLa CaSki C33A	Decrease in the angiogenic activity, induction of autophagy	Decreased expression of metalloproteinases. Inhibition of AKT and ERK1/2, destabilization of lysosomes, increased cytosol translocation	[119]
		In vitro	SiHa HeLa C33A	Inhibition of proliferation	Induction of cell apoptosis	[120]
		In vitro	HeLa	Inhibition of proliferation	Sensitization to tumor necrosis factor-related apoptosis-inducing ligand (TRAIL)	[121]
Purified Polyphenols (Curcuminoids)	Curcumin (diferuloylmethane)	In vitro	HeLa SiHa C33A	Inhibition of proliferation Induction of apoptosis	Down-regulation of HPV-18 transcription, inhibition of AP-1 binding activity and reversion of the expression of c-fos and fra-1	[122]
					Downregulation of viral oncogenes E6 and E7, NF-kB and AP-1 COX-2, iNOS and cyclin D1	[123,124,125]
					Upregulation of Bax, AIF, release of cytochrome c and downregulation of Bcl-2, Bcl-XL, COX-2, iNOS and cyclin D1	[126]
	MS17 curcumin analogue 1,5-Bis(2-hydroxyphenyl)-1,4-pentadiene-3-one	In vitro	HeLa CaSki	Cytotoxic, anti-proliferative and apoptosis-inducing potential.	Apoptosis through activation of caspase-3 in CaSki cells. Down-regulation of HPV18 and HPV16 E6 and E7 oncogene expression.	[127]
Purified Polyphenols (Lignans)	Methylenedioxy lignan	In vitro	HeLa	Inhibition of proliferation and apoptosis	Apoptosis and inhibition of telomerase activity	[128]
	Nor-dihydro-guaiaretic acid	In vitro	SiHa	Promotion of apoptosis	Downregulation of HPV E6 and E7 transcription and expression	[129]
Purified Diterpenoids	Tanshinone IIA	In vitro	HeLa SiHa CasKi C33A	Inhibition of growth promotion of apoptosis	Downregulation of HPV E6 and E7 expression, cell cycle arrest	[130]
Purified Alkaloids	Berberine	In vitro	HeLa	Inhibition of growth	Reduced expression of E6 and E7 with increase in p53 and pRb expression, loss of telomerase protein, hTERT	[131]
			SiHa		Alter epigenetic modifications and disrupt microtubule network by targeting p53	[132]
Purified Steroid Lactones	Withaferin A	In vitro	CasKi	Promotion of apoptosis	E6 and E7 repression	[133]
Purified Pyranocoumarin compounds	Decursin Decursinol	In vitro	Hela	Promotion of apoptosis	Induction of TRAIL expression	[134]
Polysaccharides fractions	From *Solanum nigrum*	In vitro and in vivo	U14	Promotion of apoptosis and inhibition of tumor growth	CD4^+^/CD8^+^ ratio modification	[135]
Lectins	From *Astragalus mongholicus*	In vitro	HeLa	Promotion of apoptosis and Antiproliferation	Upregulation of p21 and p27 and reduction of active complex cyclin E/CDK2 kinase	[136]
Peptide fractions	From *Triticum aestivum*	In vitro	HeLa	Antiproliferation	Induction of DNA damage and G2 arrest, inactivation of the CDK1-cyclin B1 complex and increase of active chk1 kinase expression	[137]
	From *Abrus precatorius*				Induction of apoptosis; generation of ROS, decrease of Bcl-2/Bax ratio, induction of mitochondrial permeability transition	[138]
Extracts	Fractionated extract of *Bryophyllum pinnata* (Bryophyllin A) Crude extract of *Phyllanthus emblica* fruits and *Brucea javanica* oil emulsion	In vitro	Hela SiHa Casky	Anti-HPV activities	Inhibitory action on AP-1 and STAT3 and specific downregulation of expression of viral oncogenes E6 and E7	[139,140,141]
	Lipid-soluble Rhizome extract of *Pinellia pedatisecta*	In vitro	Caski HeLa HBL-100	Promotion of apoptosis	Increased expression of Caspase-8, Caspase-3, Bax, p53, p21	[142]
	Basant (curcumin, purified saponins, extracts of *Emblica officinalis*, *Mentha citrata* oil, and gel extracts of *Aloe vera*)	In vitro	HeLa	Anti-HPV activities	Inhibition of transduction of HPV16 pseudovirus	[143]
	*Cudrania tricuspidata* stem extract	In vitro	SiHa HaCaT human normal keratinocytes	Apoptosis induction and cytotoxic effects in cervical cancer cells with no cytotoxic effect on HaCaT keratinocytes at concentrations of 0.125–0.5 mg/mL.	Dose-dependent mechanism by down-regulation of the E6 and E7 viral oncogenes. Apoptosis induction exclusively based on the increase of mRNA expression of extrinsic factors (i.e., Fas, death receptor 5 and TRAIL) and on activation of caspase-3/caspase-8 and cleavage of poly (ADP-ribose) polymerase (PARP)	[144]
	*Ficus carica* fruit latex	In vitro	CaSki HeLa	Inhibition of growth and invasion	Downregulation of the expression of p16 and HPV onco-proteins E6, E7	[145]

**Table 2:** Plant phytochemicals with adjuvant activity in HPV chemo- and radio-therapy.

Compound Type	Phytochemicals	Study Type	Cell Type	Activity	Mechanism of Action	References
Purified phytochemical with adjuvant activity in HPV chemo-therapies	Curcumin (diferuloylmethane)	In vitro	HeLa	Sensitization to cisplatin, paclitaxel	Induction of apoptosis by down-regulation of NF-kB.	[153]
	Tetrahydrocurcuminoids	In vitro	Drug-resistant human cervical carcinoma cell line KB-31 and KB-V-1	Sensitization to vinblastine, mitoxantrone, and etoposide	Down-regulation of HPV-18 transcription, inhibition of AP-1 binding activity, and reversion of the expression of *c*-fos and fra-1	[124]
	Quercetin	In vitro	HeLa	Sensitization to cisplatin	Enhancement of cancer cells death levels.	[154]
	Saikasaponins	In vitro	HeLa SiHa	Sensitization to cisplatin	Reactive oxygen species generation	[155]
	Wogonin (5,7-dihydroxy-8-methoxyflavone)	In vitro	A549 HeLa	Sensitization to cisplatin	Reactive oxygen species generation	[156]
	Apigenin	In vitro	HeLa SiHa	Sensitization to paclitaxel	Apoptosis through intracellular ROS accumulation	[157]
	Formonetin	In vitro	HeLa	Sensitization to epirubicin	Potentiates epirubicin-induced apoptosis via ROS production.	[158]
	Tea polyphenols with EGCG	In vitro	SiHa	Sensitization to bleomycin	Activation of caspase-3, -8, -9, and up-regulation of the expression of P53 and Bcl-2	[159]
Purified phytochemical with adjuvant activity in HPV radio-therapies	Resveratrol	In vitro	HeLa SiHa	Increased radiosensitivity and potentiation of apoptosis	Dose-dependent alteration of cell cycle progression and cytotoxic response	[160]
	Genistein	In vitro	CaSki Me180 Human esophageal cancer cell lines	Increased radiosensitivity and potentiation of apoptosis	Inhibition of Mcl-1; G(2)M arrest, and activation of the AKT gene	[161,162,163]
	Curcumin	In vitro	HeLa SiHa	Increased radiosensitivity and potentiation of apoptosis	ROS-dependent mechanism	[164]
	Ferulic acid	In vitro	HeLa Me180	Increased radiosensitivity and potentiation of apoptosis	ROS-dependent mechanism	[165]
	Quercetin	In vitro and in vivo	DLD1 (human colorectal cancer xenografts) HeLa MCF-7	Increased radiosensitivity and potentiation of apoptosis	Time- and dose-dependent mechanism and through ROS modulation and downregulation of E6 and E7 expression	[166]

**Table 3:** Plant phytochemicals and extracts in relation with their effects on HPV cancer evaluated in clinical trial settings.

Compound Type	Phytochemicals	Disease Stage	Route	Activity	References
Green tea compounds	200 mg EGCG+/− “Poly E” (37 mg epigallocatechin + 31 mg epicatechin)	51 patients with Chronic cervicitis mild dysplasia moderate dysplasia severe dysplasia	Orally (capsule) ± vaginally (ointment)	20/27 patients (74%) under poly E ointment therapy showed a response. 6/8 patients (75%) under poly E ointment + poly E capsule therapy showed a response, 3/6 patients (50%) under poly E capsule therapy showed a response. 6/10 patients (60%) under EGCG capsule therapy showed a response. Overall, a 69% response rate (35/51) was noted for treatment with green tea extracts, as compared with a 10% response rate (4/39) in untreated controls (*p* < 0.05).	[169]
Curcumin-based	Curcumin	Phase I clinical testing 4 cervical intraepithelial neoplasia (CIN) cases	Oral administration of 0.5–12 mg for 3 months	Clinical safety (up to 8 mg/day Histological improvements in 1/4 patients.	[170]
	Basant	Phase I/II double-blind clinical trial in women infected with HPV but without high grade CIN	Intra-vaginal application of curcumin-containing capsules or Basant cream	Higher clearance of cervical HPV infection (87.7%) in case of Basant cream 81.3% rate in the case of curcumin capsules compared to the controls (73.3%) with no serious adverse events.	[152]
		11 women infected with HPV and low grade cervical abnormalities	Intra-vaginal application of Basant capsules	Clearance of HPV16 infection in all the patients (11/11)	[143]
Neem-based	Praneem	20 HPV-infected women +/− early cervical intraepithelial lesions (placebo controlled)	Thirty days intra-vaginal application of Praneem tablets	Clearance of HPV16 infection in 60% of the patients (6/10). Clearance in another 50% after another administration, total 80% HPV clearance rate.	[171]
